# Employing CRISPR/Cas Technology for the Improvement of Potato and Other Tuber Crops

**DOI:** 10.3389/fpls.2021.747476

**Published:** 2021-10-26

**Authors:** Dilnur Tussipkan, Shuga A. Manabayeva

**Affiliations:** Plant Genetic Engineering Laboratory, National Center for Biotechnology, Nur-Sultan, Kazakhstan

**Keywords:** double-stranded DNA breaks, genome editing, sequence-specific nucleases, CRISPR/Cas system, tuber crops

## Abstract

New breeding technologies have not only revolutionized biological science, but have also been employed to generate transgene-free products. Genome editing is a powerful technology that has been used to modify genomes of several important crops. This review describes the basic mechanisms, advantages and disadvantages of genome editing systems, such as ZFNs, TALENs, and CRISPR/Cas. Secondly, we summarize in detail all studies of the CRISPR/Cas system applied to potato and other tuber crops, such as sweet potato, cassava, yam, and carrot. Genes associated with self-incompatibility, abiotic-biotic resistance, nutrient–antinutrient content, and post-harvest factors targeted utilizing the CRISPR/Cas system are analyzed in this review. We hope that this review provides fundamental information that will be useful for future breeding of tuber crops to develop novel cultivars.

## Introduction

### The Cell Repair Mechanism of Double-Strand DNA Breaks

Cells have various inherent mechanisms for the repair of double-stranded DNA breaks (DSBs), which are caused by endogenous and exogenous genotoxic agents (Wang and Xu, 2017; Vats et al., 2019). Cell type, cell state, and nature of the DSBs influence multiple DNA repair mechanisms. The failure and misrepair of DSBs can result in mutations and occur genome instability, and even can lead to cell death (Mcvey and Sang, 2008). However, during some essential physiological processes, DSBs are used to generate genetic diversity, such as the immune repertoire generation and the recombination of alleles during meiosis (Le Guen et al., 2015). Moreover, the loss of genetic diversity would results in an evolutionary dead end. So the DNA repair mechanism needs to cause active maintenance of genome stability and also generate genetic diversity in populations. Therefore, the high accuracy of DNA repair processes is an important phenomena in all organisms (Guirouilh-Barbat et al., 2014).

The DSB repair uses three general type of strategies. Non-homologous end joining (NHEJ) is generally regarded as error-prone, and results in mutations such as smaller insertions, deletions, and substitutions at the DSB site. It is the dominant and most active pathway in eukaryotes for repairing DSBs in all phases of the cell cycle (Anzalone et al., 2020). Homologous recombination (HR) is error-prone often with point and fragment mutations, insertions, and deletions (Rodgers and McVey, 2016). It is believed that HR is the foundation of genome engineering. In HR, the identical sister chromatids are served as templates to repair DSBs in cells during the late S/G2 phases of the cell cycle (Wang and Xu, 2017; Afzal et al., 2020). The HR, in theory, can lead to knock-in, protein-domain swapping, new gene functions, or alteration in gene regulation. HR is commonly considered as an error-free process, whereas NHEJ is considered to be error-prone process. However, Bétermier et al. (2014) and Guirouilh-Barbat et al. (2014) concluded that HR and NHEJ have a double effect, which on the one hand, they are essential for genomic stability maintenance and diversity, but on the other hand can damage the maintenance of genomic integrity. Microhomology-mediated end joining (MMEJ) is an error-prone repair system that repairs DNA breaks through using of substantial microhomologous sequence (4–25 bp) and always lead to small insertions or deletions (Mcvey and Sang, 2008; Uranga et al., 2021). The MMEJ is an active repair system during the G1and early S phases of the cell cycle (Taleei and Nikjoo, 2013).

### Genome Editing

Genome editing (GE), a recent technological innovation in the life sciences, is an example of techniques that are used to explore biological phenomena (Vats et al., 2019). In this technique, DNA is modified through cell-repair mechanisms that introduce precise breaks in the genome at specific sites (Sun et al., 2016). Genome-edited plants are not similar to conventional transgenic plants, as they may not incorporate foreign DNA. This distinction makes genome editing a unique and powerful breeding tool that has promising applications in agriculture development, especially when genome-edited crops are not adjusted as genetically-modified (GM) plants. This technique requires the introduction of sequence-specific nucleases (SSNs) into plants through the following three major methods of transformation: *Agrobacterium*-mediated transformation (Zhang et al., 2020), biolistic-mediated transformation (Hamada et al., 2018; Rustgi and Luo, 2020), and polyethylene glycol (PEG)-mediated protoplast transfection (Sant’Ana et al., 2020). Potato and most tuber crops are members of the dicotyledonous family and the most popular method for genetic transformation of dicotyledonous plants is *Agrobacterium*-mediated gene transfer.

From both a scientific and a regulatory perspective, it is beneficial if the integration of DNA in the edited plant genome is avoided (Andersson et al., 2018). In general, several strategies have been developed for obtaining transgene-free CRISPR/Cas9-edited tuber crops. They are the selection of transgene-free genome-edited plants by Mendelian segregation in T1 generation of potato obtained by using *Agrobacterium*-mediated delivery method (Ye et al., 2018; Enciso-Rodriguez et al., 2019) CRISPR/Cas9 cytidine base editor through the *Agrobacterium*-mediated delivery method (Veillet et al., 2019b), transient expression of CRISPR/Cas9 construct by the PEG-mediated transfection method (Andersson et al., 2017; Chatukuta and Rey, 2020), and ribonucleoprotein (RNP)-based genome editing (Andersson et al., 2018; González et al., 2020). In the case of Mendelian segregation, one of the limiting factors is the need of hybridization and selection of transgene-free plants from T1 segregating generations which are time consuming and laborious. The alternative delivery methods, including biolistic, PEG-mediated protoplast transfection, and RNP- based methods have been considered to be easy ways to result in non-transgenic mutants. However, these approaches always show high sensitivity and are limited to some species due to bottlenecks in the regeneration process.

Genome editing provides agriculture productivity for the world population, which has reached 7.8 billion and is estimated to exceed 10 billion by 2055 (FAO, 2017; Li and Yan, 2020). Up until 2018, GE crops have been planted approximately 191.7 million acres throughout 26 countries and adopted by approximately 17 million farmers (Yu et al., 2019; Przezbórska-Skobiej and Siemiński, 2020). The legal status between genetically modified organisms (GMO) and genome-edited products is still in discussion. However, the United States and Japan have regulatory systems at the highest political levels, whereas European countries adopted a strict policy toward genome-edited products (Menz et al., 2020). So that scientists are giving evidence that genome editing is different from genetic modification because the DNA editing method is the same as the conventional breeding method or natural biological evolution (Turnbull et al., 2021).

### Genome Editing With Sequence-Specific Nucleases

Current advancement of SSNs, such as meganuclease I-SceI (1944), zinc-finger nucleases (ZFNs,1996–2003), transcription activator-like effector nucleases (TALENs, 2009–2010), and Cas nuclease (160 kDa protein, 2013-present) offer alternative approaches for trait improvement in crop plants (Butler et al., 2015; Schiml et al., 2015; Neela and Fanta, 2019; Turk, 2019). All SSNs have been widely used in the genome editing studies for trait improvement of various plant species such as *Arabidopsis*, tobacco, and maize (reviewed in Zhang et al., 2018).

### Zinc-Finger Nucleases

Zinc-finger nucleases consist of two functional fused domains, including a non-specific DNA-cleavage domain and a DNA-binding domain composed of Cys_2_His_2_ zinc fingers, which target three base pairs and a non-specific catalytic domain of the FokI endonuclease (Bibikova et al., 2002; Nargesi et al., 2021). ZFNs were first used in model plant *Arabidopsis thaliana* (Lloyd et al., 2005). Since then, ZFNs has become increasingly popular for the genome modifications in various crop species, such as rice (Cantos et al., 2014) and maize (Gao et al., 2010). However, ZFNs have several limitations, including complexity of the contact between zinc fingers and DNA, an inherent drawback, and challenges in authenticating such proteins for a particular DNA locus of context.

### Transcription Activator-Like Effector Nucleases

Similar to ZFNs, TALENs are fusions of transcriptional activator-like effectors (TALE) repeats built from arrays of 33–35 amino acid modules and the FokI restriction enzyme from the plant pathogen *Xanthomonas* (Schiml et al., 2015; Ye et al., 2018; Afzal et al., 2020). The Fok1 nuclease is a common functional part between ZFNs and TALENs. However, designing TALENs is more affordable than ZFNs (Nargesi et al., 2021). The TALENs are simpler to construct and authenticate and thus provide a cost effective and faster method of genome editing. The challenges associated with synthesis, protein design, and corroboration remain as obstacles to more extensive use in genome-editing applications (Bétermier et al., 2014).

### Clustered Regularly Interspaced Short Palindromic Repeats

Clustered regularly interspaced short palindromic repeats (CRISPR/Cas) is an essential genome-editing technique based on the type II prokaryotic adaptive immune system from *Streptococcus pyogenes* Cas9 (SpCas9) (Schiml et al., 2015; Veillet et al., 2019a). Jennifer Doudna and Emmanuelle Charpentier first adapted gene-editing by using CRISPR/Cas9 system to function in the test tube in 2012, and were awarded the Nobel Prize for a significant contribution to Chemistry in 2020. In nature, bacteria and archaea use the RNA-guided endonucleases as part of an adaptive immune system to direct the degradation foreign nucleic acids (Anzalone et al., 2020). Although SpCas9 is predominantly used for plant functional genomics various other Cas enzymes from different bacteria have been successfully utilized as an alternative for genome editing system in plants, including the *Staphylococcus aureus* Cas9 (SaCas9) (Kaya et al., 2016), *Streptococcus thermophilus* (CRISPR1, CRISPR2, CRISPR3, and CRISPR4) (Sapranauskas et al., 2011), *Francisella novicida* Cas12a (Cpf1, FnCas12a) (Wu et al., 2020), *Brevibacillus laterosporus* (BlatCas9) (Gao et al., 2020), and *Leptotrichia shahii* (LshCas13a, Cas13a) (Veillet et al., 2019a).

**FIGURE 1:**
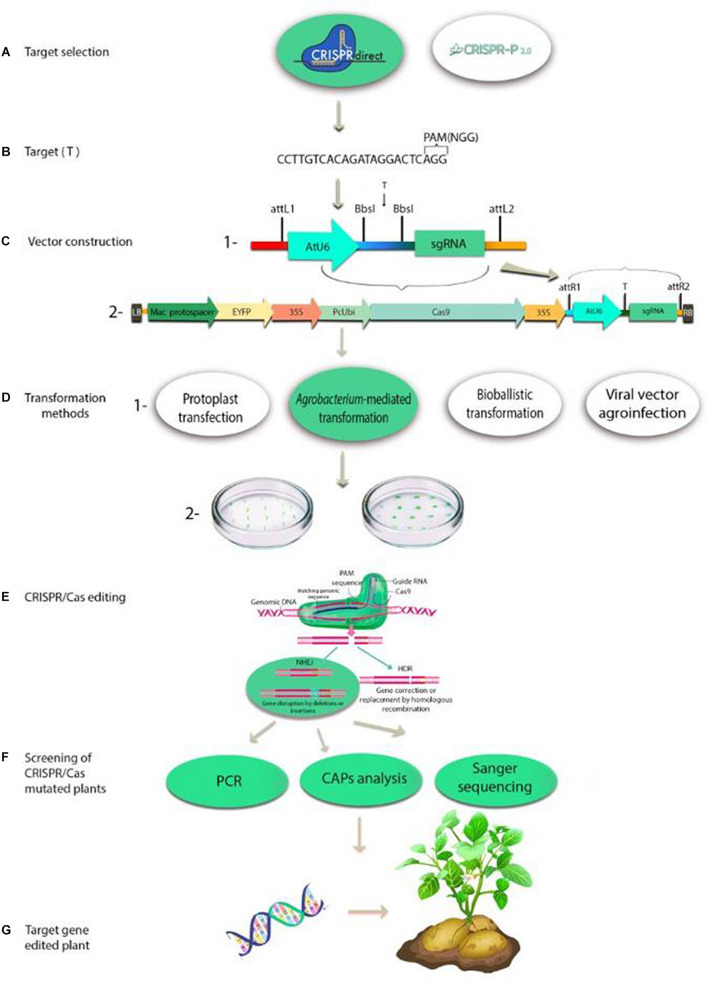
Schematic representation of plant genome editing with CRISPR/Cas: **(A)** Examples of software tools available for CRISPR sgRNA design; **(B)** The 20-bp target sequence with protospacer adjacent motif (PAM); **(C)** (1) Representation of cloning the 20-bp target sequence into the pair of BbsI sites in gRNA cloning vector; (2) Representation of cloning the gRNA expression construct into all-in-one binary vector by the recombination of attL and attR sites; **(D)** (1) Plant transformation methods are used for delivery of CRISPR/Cas9 system: protoplast transfection with polyethylene glycol (PEG) or via electroporation; *Agrobacterium*-mediated transformation; bioballistic transformation and viral vector agroinfection. (2) Examples of plant tissues used as the explants for transformation; **(E)** Diagrammatic representation of the CRISPR/Cas editing system: CRISPR/Cas induces double-stranded breaks in genomic DNA strands. The PAM sequence is primarily required for the Cas9 to cleave DNA. A stretch of 20 bases of sgRNA defines the binding specificity. Two methods repair the DSBs in DNA: non-homologous end-joining (NHEJ), which is not precise and permanently results in a gene knockout and homology-directed repair (HDR), which is activated in the presence of a template and results in knockin or gene replacement; **(F)** Screening of CRISPR/Cas mutated plants by polymerase chain reaction (PCR) analysis, cleaved amplified polymorphic sequences (CAPs) analysis and Sanger sequencing; **(G)** Target gene-edited plant in a potato example.

The CRISPR/Cas9 genome-editing system is a two-component complex composing of single-guide RNA (gRNA) and Cas9 enzyme. The customizable gRNA, a small piece of the pre-designed RNA sequence of 20 bases, binds to Cas9 and specifies a target sequence (locus) within the genome. The Cas9 enzyme acts as a pair of molecular scissor that cut the two strands of DNA at a specific location in the genome defined by the gRNA sequence, then section of DNA can be modified by adding or removing (Hongbao et al., 2018; Veillet et al., 2020). Schematic representation of plant genome editing with CRISPR/Cas is shown in Figure 1. In August 2013, three studies were published in the same issue of Nature Biotechnology showing that Cas9 works in plant cells. One of the genes considered for editing was phytoene desaturase gene (PDS3) in the model plants *A. thaliana* and *N. benthamiana* and in the crop plant *O. sativa* by transient expression system in protoplasts with the plant codon-optimized SpCas9 (pcoCas9) (Li et al., 2013; Nekrasov et al., 2013; Shan et al., 2013). In these studies other genes including flagellin sensitive 2 gene (AtFLS2) in *Arabidopsis* (Li et al., 2013), three rice genes (*OsBADH2*, *Os02g23823* and *OsMPK2*), and a wheat gene (*TaMLO*) (Shan et al., 2013) have been targeted. Higher mutation efficiency was demonstrated with the use of plant codon optimized Cas9.

The CRISPR/Cas9 system has been mainly used to generate knockout mutants by single gene editing. The development and advantage of base editors has expanded the scope of genome editing by introducing base substitutions without requiring donor DNA or induction of a DSB (Nadakuduti et al., 2018; Choi et al., 2021). Thus far, two kinds of base editors have been developed. Cytosine base editors (CBEs) catalyze the conversion of C and G base pairs to T and A base pairs, and adenine base editors (ABEs), which catalyze A and T to G and C conversions (Anzalone et al., 2020). For CBE, the Cas9 nickase (nCas9) or catalytically dead Cas protein (dCas9) is fused with a cytidine deaminase that catalyzes the deamination of cytosine (C) in an arrow window of the non-targeted strand. Deamination converts the original C to U, which is recognized as T during DNA replication, ultimately resulting in a C and G to T and A single-base substitution (Bharat et al., 2020). Base editing in the CRISPR/Cas9 system for various plant species was reviewed by Li et al. (2021). For example, Veillet et al. (2019a, 2020) developed a *S. aureus* cytosine base editor in the granule-bound starch synthase *StGBSSI* (PGSC0003DMG400012111) and *StDMR6-1* (PGSC0003DMG400000582) genes through a SanCas9 (D10A) that mediated transition (C to T) and transversion (C to G) mutations in potato.

Constant improvements in CRISPR/Cas systems have enabled more ambitious applications that aim to improve plant productivity or develop other desirable traits. One long-standing aim has been the induction of targeted chromosomal rearrangements (crossovers, inversions, or translocations) (Enciso-Rodriguez et al., 2019). Chromosome breaks in dicentric chromosomes whose centromeres are separated by >20 kb are repaired via pathways that rely mainly on sequence homology (HR, SSA) (Cook et al., 2021).

CRISPR/Cas is a flexible, simple, effective and affordable system, and it does not require any protein engineering steps in the CRISPR/Cas system construction (Amancio et al., 2017; Lino et al., 2018). As a consequence, these advantages make the CRISPR/Cas a more usable genome-editing system compare with ZFNs and TALENs. However, CRISPR/Cas still has some disadvantages and limitations, such as PAM restriction and the need for more diversity in CRISPR tools to mediate different simultaneous catalytic activities (Veillet et al., 2019a). The option of using the CRISPR/Cas tool and the general editing strategy depends on some factors such as cell type, cellular environment, a form of the agent, method of delivery each forces different constraints, and different propensities for unwanted genome modification events (Anzalone et al., 2020). All these methods including ZFNs, TALENs, and CRISPR/Cas have been applied for trait improvement in *Arabidopsis*, barley, *Brachypodium*, maize, tobacco, rice, soybean, tomato, wheat, and potato (reviewed in the references) (Malzahn et al., 2017; Nathalia and Patron, 2017; Afzal et al., 2020). The use of CRISPR/Cas9 *in vivo* and *in vitro* for crop improvement via nutritional enhancement and production of biotic and abiotic stress-tolerant plants have also been described (Afzal et al., 2020).

### Tuber Crops

Starchy root and tuber crops (RTC), including potato, cassava, sweet potato, yam, and other minor healthiest root vegetables are essential to the agriculture and food security of many countries, and are a dietary supplement of 2.2 billion people in developing countries^1^. The nutritional composition of some RTCs have been reviewed (Chandrasekara and Josheph Kumar, 2016).

Cassava, potato, and sweet potato account for 90% of the world’s tuber crop production at an annual level of approximately 836 million tons. The primary producer of the RTCs is Asia, followed by Africa, Europe, and America. In 2017, 494.6 million tons of RTC were produced worldwide (Neela and Fanta, 2019). This review covers the technological advancement, limitations, and future prospects of the CRISPR/Cas system and summarizes the application of this system in potato and other RTCs such as sweet potato (*Ipomoea batatas*), cassava (*Manihot esculenta Crantz*), yam (*Dioscorea* spp.), and carrot (*Daucus carota* subsp. *sativus*).

## Potato and Application of CRISPR/Cas System

Potato (*Solanum tuberosum* L.) is one of the most consuming crops globally after rice (Oryza s*ativa* L.), wheat (*Triticum* spp.), and maize (*Zea mays* L.) (Amir et al., 2018; Gonzales et al., 2020; Reyniers et al., 2020; Das et al., 2021). Most potato cultivars are tetraploid (2n = 4x = 48), highly heterozygous, and extremely genetically diverse with a very high single nucleotide polymorphism (SNP) frequency (Johansen et al., 2019b). Potato is grown on approximately 18.6 million ha in 150 countries, with an estimated production of 400 million tons annually (Singh, 2008). Potato occupies a wide eco-geographical range, including the northern latitudes of North America, Europe, and Asia (the long-day photoperiod) after originated in the equatorial Andean region of South America (a short-day photoperiod) (Gonzales et al., 2020). Potato grows in a temperature range of 15–29^o^C and an altitude range of 1500–3200 m a.s.l (Minda et al., 2018). The annual rainfall range requirement of the potato crop is 400–1500 mm, while the pH requirement is 5–6.5. Potato can be grown below 5 up to 4.5 pH levels.

Potatoes have potential of extraordinarily high yield and are an excellent source of more carbohydrates, proteins, minerals, and vitamins than other potential food crops (Hameed et al., 2018). These aspects have received increasing attention from scientific and many research studies have been performed on yield increase, various stress adaptation, and post-harvest conservation and processing (Sevestre et al., 2020). Unique morphological, developmental, and compositional features have been characterized for nearly 70,000 species of potato. The nuclear and organelles genome of a homozygous doubled-monoploid potato breeding line (*S. tuberosum* group Phureja DM1-3 516 R44) and a heterozygous diploid line (*S. tuberosum* group Tuberosum RH89-039-16) have been sequenced by the Potato Genome Sequencing Consortium in 2011 and predicted 39 031 protein-coding genes. This provides a platform for genetic improvement, including molecular and gene editing research in potato^2^. Historically, conventional breeding techniques have been used to increase agronomic yield, processing, and improve storage quality. Unfortunately, the conventional breeding and genetic analysis of cultivated potato has some challenging related to tetrasomic inheritance, high heterozygosity, and self-incompatibility (Nadakuduti et al., 2018). In this study, we reviewed the recent developments of CRISPR/Cas system for the potato and discuss the future perspectives (Table 1).

**TABLE 1:** Application of CRISPR/Cas system in potato (*Solanum tuberosum* L.).

Plant	Method of delivery	Technology	Target gene (s)	Trait associated with the genes	Type of mutation	Results	References
**CRISPR/Cas system for the enhancement of breeding**							
Wild type S. Chacoense, Potato (*Solanum tuberosum* L.)	*Agrobacterium-*mediated transformation; pKSE401 vector	CRISPR/Cas9 system	*S-RNase* (*S*-locus *RNase*) gene	Self-incompatibility	Single and heterozygous mutations	Self-compatibility was observed and transmitted to T1 progeny	Ye et al., 2018
Cvs. DRH-195 and DRH-310 F1 (inbred lines), Potato (*Solanum tuberosum* L.)	*Agrobacterium-*mediated transformation; pHSE40 vector	CRISPR/Cas9 system	*S-RNase* (*S*-locus *R*Nase) gene	Self-incompatibility	Bi-allelic and homozygous deletions and insertions; inversions in a biallelic configuration; Chimeric mutations; Chromosome doubling	Self-compatibility was observed and transmitted to T1 progeny	Enciso-Rodriguez et al., 2019
**CRISPR/Cas system for the enhancement of resistance biotic stress**							
Cvs. Desiree and King Edward, Potato (*Solanum tuberosum* L.)	*Agrobacterium-*mediated transformation; pDIRECT_22C vector	CRISPR/Cas9 system	*MLO* (*StMLO1*), *HDS*, AtTTM2, StDND1, StCHL1, and DMR6	Late blight	A high number of tetra-allelic deletion; very short deletions and insertions	Tetra-allelic deletion mutants showed enhanced late blight resistance	Kieu et al., 2021
Cv. Desiree, Potato (*Solanum tuberosum* L.)	*Agrobacterium-*mediated transformation; pP3, pCI, pNIb, and pCP vectors	CRISPR/Cas13a system	PVYO, PVYN, and PVYN:O; targeting the P3, CI, NIb, and CP regions.	Potato virus Y (PVY)	Not given	Lower levels of the virus PVY in systemic leaves	Zhan et al., 2019
Cv. Chicago, Potato (*Solanum tuberosum* L.)	PEG-mediated transfection method of protoplasts	CRISPR/Cas9 system	Coilin gene	Biotic stress (ordinary strain PVY-O) and abiotic (salt and osmotic) stresses.	Deletion	Higher resistance and visible differences in the edited lines F25 and D15	Makhotenko et al., 2019
Cvs. Desiree and MSX914-10 (X914-10), Potato (*Solanum tuberosum* L.)	*Agrobacterium* and GVR- mediated transformation; 35S T-DNAvector, pMDC32 and pLSL vectors	CRISPR/Cas9 system	Acetolactate Synthase1 (*StALS1*)	Herbicide Resistance	Insertion and deletion	Germline inheritance with transmission percentages ranging from 87 to 100%	Butler et al., 2015
Cvs. Desiree and MSX914-10 (X914-10), Potato (*Solanum tuberosum* L.)	*Agrobacterium* and GVR*-* mediated transformation;	CRISPR/Cas9 system; TALENs	Acetolactate Synthase1 (*StALS1*)	Herbicide Resistance	Point mutation	Reduced herbicide susceptibility phenotype	Butler et al., 2016
Tomato cv. WVA106, and cv. Desiree, Potato (*Solanum tuberosum* L.)	*Agrobacterium-*mediated transformation; CBE binary vector	CRISPR/Cas9 system	Acetolactate Synthase1 (*StALS1*)	Chlorsulfuron-resistant	Substitutions	Chlorsulfuron resistant mutants	Veillet et al., 2019b
**CRISPR/Cas system for the enhancement of resistance abiotic stress**							
Diploid clones, Potato (*Solanum tuberosum* L.)	*Agrobacterium-*mediated transformation; pJET1.2 vector	CRISPR/Cas9 system	*StCDF1* and *StFLORE*	Tuber development and drought response	Not given	Enhanced resilience drought tolerance; *StCDF1* is a non-redundant regulator of tuberization	Gonzales et al., 2020
Cv. Atlantic, Potato (*Solanum tuberosum* L.)	*Agrobacterium*-mediated transformation; VK005-14-g2 vector	CRISPR/Cas9 system	Alternative oxidase gene (*StAOX*)	High light stress	Substitutions	Inhibition of cyanide-resistant respiration.	Hua et al., 2020
**CRISPR/Cas system for the enhancement of nutrient contents in potato**							
Cv. Kuras, Potato (*Solanum tuberosum* L.)	PEG-mediated transfection method of protoplasts	Transient expression of CRISPR/Cas system	Granule-bound starch synthase (*StGBSS*)	Tuber starch quality	Insertions	Full knockout of GBSS enzyme activity	Andersson et al., 2017
Cv. Kuras, Potato (*Solanum tuberosum* L.)	RNPs delivery in protoplasts	CRISPR-Cas9 Ribonucleoproteins (RNPs)	Granule-bound starch synthase (*StGBSS*)	Tuber starch quality	Insertions	Transgene-free full knockout of GBSS enzyme activity plants	Andersson et al., 2018
Cv. Sayaka, Potato (Solanum tuberosum L.)	*Agrobacterium-*mediated transformation; pMR vector	CRISPR/Cas9 system	Granule-bound starch synthase (*StGBSS*)	Tuber starch quality	Deletions and insertions	Transformants containing dMac3 showed a higher frequency of mutations	Kusano et al., 2018
Cv. Desiree, Potato (*Solanum tuberosum* L.)	*Agrobacterium-*mediated transformation; pDIRECT_22a vector	CRISPR/Cas9 system	Starch synthase (*SS6*)	Tuber starch quality	Deletions and insertions	Deciphered function of SS6	Sevestre et al., 2020
Cvs. Desiree and Wotan, Potato (*Solanum tuberosum* L.)	PEG-mediated transfection method of protoplasts	CRISPR/Cas9 system	Granule-bound starch synthase (*StGBSS*)	Tuber starch quality	Deletions and large insertions	Increased editing efficiency of the target gene by using StU6 promotor	Johansen et al., 2019b
Cvs. Desiree and Furia, Cv. Desiree, Potato (*Solanum tuberosum* L.)	*Agrobacterium-*mediated transformation; CBE binary vector	CRISPR/Cas9 system	Granule-bound starch synthase (*StGBSS*)	Tuber starch quality	small indels,	Mediated transition (C to T) and transversion (C to G) mutations	Veillet et al., 2019a
Cv. Desiree, Potato (*Solanum tuberosum* L.)	*Agrobacterium-*mediated transformation; CBE binary vector	CRISPR/Cas9 system	Granule-bound starch synthase (*StGBSS*) and *StDMR6-1*	Tuber starch quality	Small insertions or deletions	Mediated transition (C to T) and transversion (C to G) mutations	Veillet et al., 2020
**CRISPR/Cas system for reduction of anti-nutrient contents in potato**							
Cv. Mayqueen, Potato (*Solanum tuberosum* L.)	*Agrobacterium-*mediated transformation; pMgP237-2A-GFP vector	CRISPR/Cas9 system	*St16DOX*	Steroidal glycoalkaloids (SGAs) in potato hairy roots	Deletion, Insertion	Abolition of *St16DOX* wild-type sequences in tetraploid potato	Nakayasu et al., 2018
**CRISPR/Cas system for the reduced of post-harvest factors affecting potato**							
Cv. Desiree, Potato (*Solanum tuberosum* L.)	Protoplasts	CRISPR/Cas9 system	Polyphenol Oxidases catalyze (*StPPO2*)	PPOs activity in the tuber	Small and larger deletions	Lines with a reduced PPO activity and enzymatic browning	González et al., 2020

### CRISPR/Cas System for the Enhancement of Potato Breeding

Plant genetic resources are the most essential natural resources in the world. Many crops species are recognizable as hybrids that have been domesticated from the wild type polyploidy plants or generated by various conventional or modern biotechnological breeding program (Alix et al., 2017; Bevan et al., 2017). The conventional mutagenesis-based breeding processes are time-consuming and laborious, especially for polyploid crop breeding (Liu et al., 2021). Re-domestication of diploid potatoes applying modern agricultural technologies has made good progress in unlocking the genetic diversity of potatoes. However, most diploid potato species have gametophytic self-incompatible, which has been a limiting factor for the development of inbred lines in potatoes. The genetic basis of self-incompatible mechanism in flowering plants, including the *Solanaceae* family was reviewed in the some researches (Kao and McCubbin, 1996; Silva and Goring, 2001). These technologies include genetic engineering, molecular marker technology, *cis*-genesis, and more recently gene editing (Beumer et al., 2021).

Self-incompatibility is genetically determined by the highly polymorphic S-locus, which consists of strongly linked genes, termed as the pistil-expressed *S-RNase* (S-locus *RNase*) and the pollen-expressed F-box proteins (*SLF* or S locus F-box). The pollen tube growth is one of the main self-incompatible indicators, and a multi-allelic RNase in the pistil blocks incompatible pollen tube growth. To generate self-compatible diploid potato lines, the CRISPR/Cas9 system was used to target the first and second exons of the *S-RNase* gene and self-compatibility was confirmed in T1 lines (Ye et al., 2018; Enciso-Rodriguez et al., 2019). Ye et al. (2018) revealed that a self-incompatible diploid potato could be reinvented with a heterozygous mutation by knocking out the *S-RNase* alleles, and only a single mutation was transmitted to the T1 generation. Further research revealed that self-compatibility was achieved in the *S-RNase* knockout T0 lines, which contained bi-allelic and homozygous deletions/insertions in self-incompatible genotypes, and transmitted self-compatibility to T1 progeny with a potential transgenerational deletion (Enciso-Rodriguez et al., 2019). This study also confirmed that *S-RNase* is located within a pericentromeric region of 6.1 and 18.9 Mb of chromosome 1 in the physical map.

### CRISPR/Cas System for Enhancement of Resistance to Biotic Stress

Biotic stress in plants is occurred by living organisms, commonly viruses, bacteria, fungi, nematodes, insects, arachnids, and weeds (Singla and Krattinger, 2016; Hashem et al., 2019). Potato crops are suffering several pests and diseases, such as late blight (caused by the oomycete pathogen *Phytophthora infestans*), Colorado potato beetle (*Leptinotarsa decemlineata*), bacterial wilt (caused by *Ralstonia solanacearum*), early blight (caused by the fungus *Alternaria solani*), and potato blacklegs (bacterial infection) (Kieu et al., 2021)^3^. Various pathogen infections affect potato yield with high economic losses of 30–64% every year (Torrance and Talianksy, 2020), even it is reached 88% (Kolychikhina et al., 2021). So the identification and characterization of disease resistance genes in the major crops genome have been a main focusing issue for the research community.

CRISPR/Cas has been used to enhance resistance to potato late blight, which remains the most significant disease of potato cultivation globally (Hameed et al., 2018). Late blight is a fungal disease that can destroy the leaves, stems, and tubers of potato plants (Tsedaley, 2014) and exhibits rapid multiplication and dissemination. Tiwari et al. (2021) published late blight resistance genes (*R3a*, *RGA2*, *RGA3*, *R1B-16*, *Rpi-blb2, Rpi*, and *Rpi-vnt1*) in potato. Susceptibility genes (*S*-genes) are important for increasing pathogen resistance (Tiwari et al., 2021). Potato pathogen susceptible genes *StMLO1* (Mildew locus O), *HDS* gene homologue (DMG400008050), *AtTTM2* gene homolog (DMG400025117), *StDND1* (DMG400001441), *StCHL1* (DMG400000711), and other two *DMR6* homologues (DMG400000582 and DMG401026923) were edited with CRISPR/Cas9. The mutant lines encoding *StMLO1, HDS*, and *AtTTM2* genes were as susceptible to late blight as wild type, whereas *StDND1, StCHL1*, and *StDMR6-1* tetra-allelic deletion mutants exhibited enhanced resistance to late blight (Kieu et al., 2021).

Potato virus X (PVX, genus potexvirus), potato virus Y (PVY, genus potyvirus), potato virus A (PVA), potato virus M (PVM), potato virus S (PVS), and potato leafroll virus (PLRV, genus polerovirus) have been recorded in all potato growing zones and are probably the most diverse and economically harmful viruses infecting the world’s potato production (Halabi et al., 2021). The PVY have positive-sense (+), single-stranded RNA genome, with an average size of 9.7 kb in length. Upon translation, the RNA genome encodes a single large polyprotein of around 3061 amino acid residues. The single large polyprotein is processed by three virus-specific proteases domains into 11 mature proteins, namely P1, HC-Pro, P3, P3N-PIPO, 6K1, VPg, CI, 6K2, NIa (VPg plus Pro), NIb (viral replicase), and CP (capsid protein) (Quenouille et al., 2013; Cui and Wang, 2016). Zhang et al. successfully applied the CRISPR/Cas13a system by programming sgRNA (named pP3, pCI, pNIb, and pCP) specific for the RNA genome of three PVY strains (PVY^O^, PVY^N^, and PVY^N:O^). When transgenic lines with high transgene expression levels were assessed for PVY resistance with the PVY^o^ virus, PVY mosaic symptoms were detected in leaves of infected WT plants at 25 days post-inoculation, whereas, no disease symptoms were detected in any of the transgenic plants. ELISA and qRT-PCR results indicated that transgenic plants accumulated much lower levels of the virus in systemic leaves than WT plants after PVY inoculation (Zhan et al., 2019).

One of the genes associated with plant stress is coilin. The mechanisms associated with the protective response to stress are unclear. Coilin is a major structural scaffold protein necessary for Cajal bodies, which are dynamic subnuclear compartments involved in the biogenesis of ribonucleoproteins. Makhotenko et al. (2019) applied the CRISPR/Cas9 system on the coilin gene in the potato cultivar Chicago. The edited lines (named F25 and D15) showed significantly higher resistance than control plants to biotic (ordinary strain PVY-O) and abiotic (salt and osmotic) stresses (Makhotenko et al., 2019).

Development of herbicide-resistant crops has resulted in remarkable changes to sustainable development of agronomy, one of which is the adoption of practical, effortless, and scientific crop-production systems with green technology (Vencill et al., 2012). The recent advantages of CRISPR/Cas9-mediated gene editing allows to target endogenous genes such as acetolactate synthase (ALS), 5-enolpyruvylshikimate-3-phosphate synthase (EPSPS), cellulose synthase A catalytic subunit 3 (CESA3), and splicing factor 3B subunit 1 (SF3B1) (Han and Kim, 2019). ALS, also called acetohydroxyacid synthase, is found in plants and microorganisms and catalyzes the biosynthesis of various amino acids, including valine, leucine, and isoleucine (Ay et al., 1998). Resistance to ALS inhibitors is often attributed to an amino acid substitution in ALS (Svitashev et al., 2015). One of the first applications of the CRISPR/Cas system for ALS gene editing in potato was performed by Butler and colleagues (Butler et al., 2015, 2016). The StALS1 gene of the diploid and tetraploid potato was modified using CRISPR/Cas and TALEN with transformation of a binary T-DNA vector [conventional T-DNA and modified geminivirus (GVR) T-DNA]. Transformed events modified with GVRs introduced point mutations that supported a more herbicide-resistant phenotype and inheritable mutations, while events transformed with conventional T-DNAs had no detectable mutations and had same appearance as wild type. The *ALS* gene was also successfully and efficiently targeted in tomato and potato plants by CBE using *Agrobacterium-*mediated transformation. This resulted in chlorsulfuron-resistant mutant plants, as reported transgene-free edited plants were generated in the first offspring (Veillet et al., 2019b).

### CRISPR/Cas System for Enhancement of Resistance to Abiotic Stress

The extreme levels of abiotic stresses such as drought, flooding, salinity, mineral deficiency, low or high temperatures, high light, post-harvest problems, and accumulation of reducing sugars during cold storage have negative impact in crop yield (Le Gall et al., 2015). Abiotic stresses usually lead to superoxide overproduction in mitochondria (Dourmap et al., 2019). The mitochondrial respiratory chain in higher plants consists of the ATP-coupling cytochrome pathway (CP) and the cyanide (CN)-resistant respiratory pathway. The relationship of this chain to abiotic stress has been studied (Watanabe et al., 2010; Afzal et al., 2020). Plant mitochondria have an alternative oxidase (AOX), which is encoded by a small family of nuclear genes (Saha et al., 2016). AOX catalyzes the ATP-uncoupling cyanide (CN)-resistant pathway and can command the synthesis of important signaling molecules, such as hydrogen peroxide (H_2_O_2_), superoxide (O_2_^–^), and nitric oxide (NO). AOX is present in plants, fungi, protozoa, and some invertebrates. At the beginning of the 20th century, the AOX was first discovered from thermogenic plants during anthesis (Saha et al., 2016). Later it was found that it exists in the inner mitochondrial membrane of plants, fungi, protozoa, and some invertebrates (Araújo Castro et al., 2017). AOX activity can protect cells from reactive oxygen species (ROS) such as O_2_^–^, H_2_O_2_, and OH, particularly during different abiotic stress (Wang and Vanlerberghe, 2013). A CRISPR vector for the functional gene *StAOX* of the potato cyanide-resistant respiratory pathway was designed to examine the role of cyanide-resistant respiration in potato against high-light stress. The results indicated that high-light stress induced expression of AOX and accelerated cyanide-resistant respiration, and that loss of *StAOX* directly led to inhibition of cyanide-resistant respiration (Hua et al., 2020).

CRISPR/Cas9 technology has been widely applied for plant genomics research, with a focus on not only loss-of-function and gain-of-function analysis, but also on plant gene association analysis. In the potato genome, the association of transcription factor *StCDF1* with the antisense transcript *StFLORE* to enhance drought tolerance was detected by mutation of the *StFLORE* promoter with CRISPR/Cas9 system. Although indicated that the overexpression of *StFLORE* or the downregulation of *StCDF1* increased tolerance to drought through the regulation of stomata size and number, and *StFLORE* is regulated by *StCDF1* (Gonzales et al., 2020).

### CRISPR/Cas System for Enhancement of Nutrient Content in Potato

Several studies have described improvements in the nutritional value of potato tubers, for example by enhancing essential amino acid compounds of seed protein (lysine, methionine, cysteine, and tyrosine contents), triacylglycerols, and vitamin E by overexpressing *AmA1*, *PrLeg*, *WRINKLED1, DIACYLGLYCEROL ACYLTRANSFERASE* 1, *OLEOSIN*, *At-HPPD* and *At-HPT* genes (Raina and Datta, 1992; Chakraborty et al., 2000, 2010; Crowell et al., 2008; Goo et al., 2013; Xu et al., 2020). Potato is also one of the major crops grown for starch production. Starch granules of potatoes have typical microscopic structures with different shapes and sizes, neutral taste, and good paste clarity (Singh et al., 2016). The starch content in potato, sweet potato, cassava, yam and maize endosperm consists of 65–90% of the absolute dry weight (Tigabu and Desta, 2018). Technology for starch isolation from natural sources is relatively simple and the range of industrial starch-based products is quite extensive and diverse. Starch extracted from potatoes has many utilization in food and technical-industrial applications, as potato starch has a variety of preferable molecular and intermolecular features (purity, molecular weight, phosphorylation, branching) (Khlestkin and Eltsov, 2021). Starch contains two major different carbohydrate, namely amylose (20–30%) and amylopectin (70–80%). Due to the low solubility of amylose, amylose solutions are generally unstable and easily to retrograde, gel, and turn opaque. In contrast, amylopectin is highly soluble in water, which characteristically makes amylopectin solutions very stable and clear (Visser et al., 1997). Changing the ratio of these two components significantly alters the properties of the starch. Therefore, starch has been the target of numerous genetic modification studies for a variety of purposes. Mutations in granule-bound starch synthase (*GBSS*) and two starch synthase (SS) genes (*SSII* and *SSIII*) resulted in starch with short-chain amylopectin and gelatinization (Khlestkin et al., 2017). In potato, the GBSS enzyme is encoded by a single locus (GBSSI) with four alleles in cultivated potato. Granule-bound starch synthase (GBSS) plays an essential role in amylose synthesis (Miao et al., 2014). In cultivated potatoes, the GBSS enzyme is encoded by a single locus (*GBSSI*) with four alleles. Waxy or amylose-free (amf) starch is a new type of starch made uniquely out of amylopectin molecules. Due to its superior viscosity and elasticity, amf starch has found applications in many areas of the food industry, such as stabilizers and thickeners. Waxy potato has been generated by silencing *GBSSI* gene function using traditional mutational breeding (Visser et al., 1991; Kuipers et al., 1994; Muth et al., 2010), RNAi (Andersson et al., 2003) and modern gene editing technology (Andersson et al., 2018; Veillet et al., 2019b). The *StGBSS* gene in the cultivar Kuras has been targeted for full knockout of GBSS enzyme activity using transient expression of CRISPR/Cas9 components in protoplasts, either as DNA plasmids or as ribonucleoprotein (RNP) complexes. As a result, the starch was confirmed as being of amylopectin quality in the line with all four alleles mutated (Andersson et al., 2018). In the study of Kusano, to the high-level expression of the CAS9 gene, two traditional enhancers were used for the efficient mutation of GBSSI. One of these enhancers was a translational enhancer dMac3 sequence (161 nucleotides), which consisting of a portion of the OsMac3 mRNA 5′-untranslated region (UTR). Another one was 5′-UTR from the alcohol dehydrogenase (ADH) gene. As the result, transformants containing dMac3 showed a higher frequency of mutations compare with transformants containing ADH enhancer and no enhancer sequence (Kusano et al., 2018).

Furthermore, multiple of unknown proteins associated with potato starch granules and multiple protease inhibitors were identified. Among these proteins, a still unknown isoform of starch synthase (*SS6* gene coordinates on chromosome 7) demonstrated a great potential to be a key enzyme of the starch biosynthetic pathway in potato (Helle et al., 2018). Knockout mutagenesis of SS6 resulted in deletions and the function of SS6 was deciphered. The properties of the starch generated by this enzyme has great potential in industrial applications (Sevestre et al., 2020). CRISPR/Cas9 editing efficiency may be improved by using endogenous plant-specific U6 promoters, which contributes to increased sgRNA levels. Replacement of the regularly used *Arabidopsis* AtU6-1 promoter with endogenous potato StU6 promoters resulted in enhanced editing efficiency of the GBSS gene, with editing frequencies of 30–70% in protoplasts and 35% full allelic gene editing (Johansen et al., 2019b).

### CRISPR/Cas System for Reduction of Anti-nutrient Content in Potato

Another research priority for scientists and breeders is to improve potato tuber quality by removing anti-nutritional compounds, such as steroidal glycoalkaloids, acrylamide, and food toxins (Hameed et al., 2018). Potato accumulates the poisonous and bitter-tasting solanidane glycoalkaloids α-solanine and α-chaconine. These substances are harmful to many living things (Akiyama et al., 2021). It is known that *St16DOX* is the single gene responsible for steroid 16α hydroxylation in SGA biosynthesis and is an ideal research objective for generation CRISPR/Cas9-mediated mutagenesis to SGA-free potato plants. The knockout of *St16DOX* gene resulted in multiple mutations, including chromosomal fragment deletion at different sites, which resulted in abolition of St16DOX wild-type sequences in tetraploid potato (Nakayasu et al., 2018).

### CRISPR/Cas System for the Reduced of Post-harvest Factors Affecting Potato

The post-harvest storage of potato tubers is also one of the key factors that determine the processing quality and final products. This long-term storage always accompanies with various storage diseases, such as soft rot, black dot, and *Fusarium* dry rot, which significantly affect tuber quality and makes it unsuitable for further processing (Barley Disease Control, 2009; Hameed et al., 2018). Potatoes are usually subjected to cold storage (4–8^o^C) to achieve a more sustainable supply to consumers throughout the year. However, cold temperature increases the reducing sugars accumulation in potato tubers. Upon high-temperature processing, these reducing sugars react with free amino acids to produce a brown and bitter-tasting products, and elevated levels of acrylamide, which is considered a potential carcinogen (Clasen et al., 2016). Enzymatic browning of potatoes is a major problem that arises during harvest and post-harvest procedures such as transport, storage, distribution, and blanching. In enzymatic browning, polyphenol oxidases (PPOs) catalyze the rapid polymerization of o-quinones from natural phenols. As a result, the chemical reaction leads to the formation of dark-colored precipitates in fruits and vegetables with negative effects on color, taste, flavor, and nutritional value (Yoruk and Marshall, 2010).

Four genes are mainly responsible for PPO activity in the potato tuber. The *StPPO2* (PGSC0003DMG400018916) gene is the principal contributor to PPO total protein content (55% of the total enzyme), followed by *StPPO1* (PGSC0003DMG400029575; 25–30%) and *StPPO4* (PGSC0003DMG400018917) and *StPPO3* (PGSC0003DMG400018914) (both < 15%). According to some reports, the down-regulation of multiple *StPPO* genes may harm the other functions of the enzyme in the plant. González et al. (2020) developed a CRISPR/Cas-based genome-editing approach to target *StPPO2*. Mutations induced in the four alleles of *StPPO2* gene showed a significant reduction (up to 69%) in tuber PPO activity and enzymatic browning of (73%) in compared to the control (González et al., 2020). In this study, we also summarized CRISPR/Cas studies for other tuber crops and discuss the future perspectives (Table 2).

**TABLE 2:** Application of CRISPR/Cas system in sweet potato (*Ipomoea batatas*), cassava (*Manihot esculenta Crantz*), yam (*Dioscorea* spp.), and carrot (*Daucus carota subsp. sativus*).

Plant	Method of delivery	Technology	Target gene (s)	Trait associated with the genes	Type of mutation	Results	References
**Sweet potato (*Ipomoea batatas*)**							
Cvs. Xushu22 and Taizhong6, Sweet potato (*Ipomoea batatas*)	*Agrobacterium-*mediated transformation; *IbSBEII-sgRNA2* and *IbSBEII-sgRNA12* vectors	CRISPR/Cas9 system	Granule-bound starch synthase and starch branching enzyme (*IbGBSSI* and *IbSBEII*)	Tuber starch quality	Deletions, substitutions, and insertions	Knockout of *IbGBSSI* reduced amylopectin, while the knockout of *IbSBEII* increased the amylose percentage	Wang et al., 2019
**Yam (*Dioscorea* spp.)**							
Cv. *D. alata*, Yam (*Dioscorea* spp.)	*Agrobacterium-*mediated transformation	CRISPR/Cas9 system	Phytoene desaturase gene (*DrPDS*)	Albino	Deletion and insertion	Promoter DaU6.5 performed best, while DaU6.2 and DaU6.3 yielded similar efficiency for expressing gRNAs	Syombua et al., 2021
**Cassava (*Manihot esculenta*)**							
Model cv.60444, Cassava (*Manihot esculenta*)	*Agrobacterium-*mediated transformation; pCAMBIA2300 vector	CRISPR/Cas9 system	Novel cap-binding protein (*nCBP*-1 and *nCBP*-2) genes	Cassava brown streak disease (CBSD)	Deletions and insertions	Reduced CBSD severity in root and stem	Gomez et al., 2018
Cassava (*Manihot esculenta*)	*Agrobacterium*-mediated transformation; pJET1.2 vector	CRISPR/Cas9 system	African cassava mosaic virus genes (*AC2* and *AC3*)	African cassava mosaic virus	Single-nucleotide insertion, and substitution	Resulted in the lack of resistance	Mehta et al., 2019
Cvs. model cv.60444, *M. esculenta* T200 and *M. esculenta* TME3, Cassava (*Manihot esculenta*)	PEG-mediated transfection method of protoplasts	CRISPR/Cas9 system	Ubiquitin E3 ligase gene (*MeE3L*)	South African cassava mosaic virus (SACMV)	Single-nucleotide insertion	Accumulation of SACMV DNA was increased in the mutant lines	Chatukuta and Rey, 2020
**Carrot (*Daucus carota* subsp. *Carota)***							
Carrot (*Daucus carota* subsp. *Carota*)	*Agrobacterium*-mediated transformation; pYPQ154, pYPQ166, and pYPQ167	CRISPR/Cas9 system	*F3H*	Anthocyanin compound	Small Indels, and long chromosome fragment deletions	Individual transgenic calli, which differing in degree of discoloration from white, mosaic to purple. No transgenic plant was regenerated	Klimek-Chodacka et al., 2018
Cvs. Kurodagosun and Deep purple, Carrot (*Daucus carota* subsp. *Carota)*	*Agrobacterium-*mediated transformation; *VK005* vector	CRISPR/Cas9 system	*DcPDS* and *DcMYB11*3-like genes	Albino and purple depigmentation	Insertion, deletion, and substitution	Generated Albino-type and purple depigmented plants	Xu et al., 2019

## Sweet Potato, Yam, Cassava, Carrot, and Application of CRISPR/Cas System

### Sweet Potato

Sweet potato (*Ipomoea batatas*) is one of the main starch-rich tuber crops and is a source of carbohydrates, β-carotene, vitamin A, C, B, and E complex, calcium, and iron. Cultivated forms of sweet potato have 2n = 6x = 90 chromosomes and are widely grown in 111 countries, particularly Southeast Asia is the major contributor, following by Oceania, and China. Sweet potato originated from either the Central or South American lowlands (Saranraj et al., 2019). Based on nutritional qualities and sensory acceptability, the color of sweet potato flesh varies from white, yellow, purple, and orange (Neela and Fanta, 2019). In contrast to other staple food crops, sweet potato possess special attributes, such as adaptability to wider topography, good productivity in short duration, and balanced nutritional composition (Trancoso-Reyes et al., 2016). To improve starch quality in sweet potato, the genes encoding starch biosynthesis (*IbGBSSI*- and *IbSBEII*) were subjected to CRISPR/Cas9-based mutagenesis by Wang et al. (2019). Most of mutations were nucleotide substitutions that cause amino acid changes and, less frequently, to stop codons. This study demonstrated the efficiency of CRISPR/Cas9 technology for improvement of starch qualities in sweet potato (Wang et al., 2019).

### Yam

Yam (*Dioscorea* spp.) is a multi-species tuber crop that supplies food and income to millions of people around the world, particularly in Africa. The “yam belt” in West Africa includes Nigeria, Benin, Togo, Ghana, and Cote d’Ivoire and produces 92% of the 72.6 million tons of worldwide global yam production (NARIT; http://narit.or.th/images/category/dioscorea-spp-yam-4f349d). Yams are consumed raw, as a cooked soup, or used as a powder and flour in food preparations. Yam tubers are not only considered as a fundamental carbohydrate with relatively high protein and ascorbic acid content, but also have many bioactive components, such as mucin, dioscin, dioscorin, allantoin, choline, polyphenols, diosgenin, and vitamins such as carotenoids and tocopherols (Iwu et al., 1990). The phytoene desaturase gene (PDS) is essential for chlorophyll biosynthesis and is involved in converting phytoene into the carotenoid precursors phytofluene and carotene (Mann et al., 1994). PSD knockout mutations have an albino phenotype and thus are usually used as a visual marker to approve genome editing in various plants, including rice (Banakar et al., 2020), *Arabidopsis, M. truncatula*, *N. benthamiana* (Li et al., 2013; Wolabu et al., 2020), and potato (Butler et al., 2020). The CRISPR/Cas9 system was successfully used to target the PDS gene in yam. The efficiency of yam-derived promoters was identified by expression of gRNAs for PDS gene editing. Promoter DaU6.5 performed the best efficiency, while DaU6.2 and DaU6.3 had similar efficiency as revealed by fluorescence scores (Syombua et al., 2021).

### Cassava

Cassava (*Manihot esculenta*) is one of the most major staple crops for around 800 million people in tropical and sub-tropical regions of the world (Alicai et al., 2016). Africa presents over 50% of the world cassava production of 233.8 million metric tons. Cassava brown streak disease (CBSD) is the most devastating cassava disease in the Eastern, Central, and Southern Africa. Nowadays, West Africa including Nigeria, the largest cassava producer in the world, is also suffering from this disease (Alicai et al., 2019).

Cassava brown streak disease is caused by two species of positive-sense RNA viruses belonging to the family Potyviridae and genus Ipomovirus, specifically CBSV and Ugandan cassava brown streak virus (UCBSV) (Winter et al., 2010). The CBSV genome contains a polyprotein of 2902 amino acids that is proteolytically cleaved into 10 mature proteins (Mbanzibwa et al., 2009). Diseases caused by the family Potyviridae require the interaction of viral genome-linked protein (VPg) and host eukaryotic translation initiation factor 4E (eIF4E) isoforms. The eIF4E protein family assumes a fundamental par in the initiation of cap-dependent mRNA translation. It is known that cassava encodes the five eIF4E proteins such as eIF4E, eIF(iso)4E-1, eIF(iso)4E-2, novel cap-binding protein-1 (nCBP-1), and nCBP-2. Protein–protein interaction experiments consistently demonstrate that VPg proteins associate with cassava nCBPs. CRISPR/Cas9-mediated genome editing was applied to generate ncbp-1, ncbp-2, and ncbp-1/ncbp-2 mutants in cassava cultivar 60444. Compared to wild-type, the ncbp-1/ncbp-2 double mutants showed more resistance to CBSD severity in root and stem (Gomez et al., 2018). Among the viruses that infect cassava, cassava mosaic disease (CMD) is the most dangerous and widespread in the cassava-growing field. At least seven cassava mosaic geminivirus (CMG) species, including South African cassava mosaic virus (SACMV) limit crop production in Africa (Patil and Fauquet, 2010). The complete nucleotide sequences of infectious clones of SACMV were determined and this enabled the selection of putative host interacting genes for different investigations (Berrie et al., 2001). CRISPR/Cas9 technology was used in cassava with the aim of engineering resistance to SACMV. The sgRNAs of AC2 gene that is coding for the multifunctional TrAP protein and AC3 gene that is coding for the REn protein were targeted, but a clear disease-resistance phenotype was not observed in mutant lines (Mehta et al., 2019).

Plant gene association analysis as described above in potato plants has also been applied in cassava. For example, the association of SACMV with the ubiquitin E3 ligase gene (*MeE3L*) was studied by CRISPR/Cas9-mediated system of cassava gene *MeE3L* in SACMV-infected cassava protoplasts (Chatukuta and Rey, 2020).

### Carrot

Carrot (*Daucus carota* subsp. *carota* L.) is an economically important toot vegetable crop. Carrots are grouped into the carotene or western (*D. carota* ssp. *sativus* var. sativus) and the anthocyanin or eastern groups (*D. carota* ssp. *sativus* var. atrorubens Alef.). Purple carrot cultivars accumulate rich cyanidin-based anthocyanins, while orange, yellow, and red carrot cultivars accumulate rich carotenoids in the taproots (Xu et al., 2017). Carotenoids and anthocyanins both provide various health benefits to humans. Anthocyanins are an enormous group of more than 500 pigments abundant in the plant kingdom that accumulate in the cell vacuole of certain some organs. Many of the genes that encode enzymes involved in anthocyanin biosynthesis have been well characterized. For the first time, CRISPR/Cas9-mediated loss of function of the gene F3H, which is critical for anthocyanin biosynthesis, was reported by Klimek-Chodacka et al. (2018). They studied three codon-optimization variants of SpCas9 genes and demonstrated that AteCas9 has high efficiency in carrot cells for producing mutations, followed by zCas9, and Cas9p. The knockout of F3H gene affected the discoloration of calli, however, no transgenic plant was regenerated.

Albino and purple depigmentation mutants were regenerated when DcPDS and DcMYB113-like genes in orange and purple carrot were targeted by CRISPR/Cas9, respectively (Xu et al., 2019). The function of PDS genes is described above, while the DcMYB113-like changes critical functions in anthocyanin biosynthesis, and knockout mutations were expected to cause depigmentation phenotype. In addition, four different promoters (AtU3b, AtU3d, AtU6-1, and AtU6-29), which individually drive four sgRNA expression cassettes, were tested for mutation efficiency rate. The highest efficiency of mutagenesis was noticed in the loci targeted by AtU6-29-driven sgRNAs in both *DcPDS* and *DcMYB113*-like knockout T0 plants (Xu et al., 2019).

## Conclusion

CRISPR/Cas-mediated genome editing technology can selectively modify any part of a genome to target genes controlling stress tolerance and nutritional quality of tuber crops. The simplicity and versatility of this technology make it a powerful tool for precise crop improvement via generation of knockout mutations in the form of insertions, deletions, and substitutions. The development of base editors, which generate base substitutions without requiring donor DNA or DSB induction, has expanded the possibilities of genome editing. The cultivated potato and most tuber crops have a complex genetic structure due to autotetraploidy and heterozygosity. Genetic improvement of these crops presents numerous challenges using traditional breeding techniques. To this end, the genome sequences of potato and other tuber crops provides a platform for genetic improvement via gene-editing technology. As mentioned above the CRISPR/Cas9 system with increased mutagenesis frequency has been established by using the improved CRISPR/Cas9 vector containing a translational enhancer sequence dMac3 in potato (Kusano et al., 2018) and through the use of codon optimized U6 promoter according species (Johansen et al., 2019a; Syombua et al., 2021).

Although significant progress in the last 5 years has made it possible to increase the efficiency and target specificity of CRISPR technology by using marker gene such as PDS, more work remains to be done to improve this technology for agriculturally important traits of commercial cultivars. In potato and other tuber crops, it is possible use the fluorescence marker-assisted selection for transgene-free genome-edited plants by the co-expression of fluorescence marker genes, as has been done for rice, tomato, and Arabidopsis (Aliaga-Franco et al., 2019). All the more already investigated the potentials of green fluorescent protein as a marker to select transgenic hybrid potato tubers (Palumbo and Veilleux, 2007) and red fluorescent protein to identify the localization of key enzymes of carotenoid biosynthesis after stable transformation in potato (tubers) (Nick, 2013). However, the fluorescence marker facilitates them of the transgene-free plants, but it does not improve the ratio of transgene-free plants. The self-eliminating and Transgene Killer CRISPR system proposed by He et al. (2018) and He and Zhao (2020) may accelerate identification of transgene-free and CRISPR-edited plants. For that they have modified the CRISPR/Cas9 construct with the bacterial BARNASE gene under the control of the rice REG2 promoter to express the suicide transgene during early embryo development to kill all of the CRISPR/Cas9-containing pollen and embryos produced by T0 rice plants that can be used for tuber crops in perspective.

One of the main limiting factors is the low embryogenic competency of local crops to tissue culture. It would be relevant to use the CRISPR/Cas technology to control endogenous plant hormones controlling and triggering plant morphogenesis, including somatic embryogenesis and organogenesis. Another major limiting factor is the size of Cas9, which prevents the use of plant viral vectors to deliver CRISPR/Cas components into a plant genome. Further research in this direction would widespread use the CRISPR/Cas technology to improve agro-important traits of local crop cultivars.

The studies reported in this review indicate that genome improvement in existing potato and other tuber crops is possible with this technology. The use of CRISPR/Cas for gene knockouts and base-editing systems for expression regulation of any gene of interest will facilitate development of non-transgenic crops. Recent developments in CRISPR-based genome editing technology for analyzed crops allow not only targeting of single genes in the genome, but also modification of multiple genes at once. One of the problems was observed that is utilization of genome-editing technology for model cultivars, due to the low regeneration efficiency of local cultivars in tissue culture. By considering the use of CRISPR/Cas technology as reported in this review, scientists may be able to design efficient vectors and transformation systems for employing CRISPR/Cas technology in tuber crops.

## Author Contributions

TD and SM wrote the manuscript together. Both authors contributed to the article and approved the submitted version.

## Conflict of Interest

The authors declare that the research was conducted in the absence of any commercial or financial relationships that could be construed as a potential conflict of interest.

## Publisher’s Note

All claims expressed in this article are solely those of the authors and do not necessarily represent those of their affiliated organizations, or those of the publisher, the editors and the reviewers. Any product that may be evaluated in this article, or claim that may be made by its manufacturer, is not guaranteed or endorsed by the publisher.

## Footnotes

1
https://www.nri.org/development-programmes/root-and-tuber-crops-in-development/overview

2
https://www.nature.com/articles/nature10158#citeas

3
https://cipotato.org/potato/potato-pests-diseases/
